# Clinical genetics in transition—a comparison of genetic services in Estonia, Finland, and the Netherlands

**DOI:** 10.1007/s12687-021-00514-7

**Published:** 2021-03-11

**Authors:** T. Vrijenhoek, N. Tonisson, H. Kääriäinen, L. Leitsalu, T. Rigter

**Affiliations:** 1 Department of Genetics, University Medical Centre Utrecht, Utrecht, The Netherlands; 2 Estonian Genome Centre, Institute of Genomics, University of Tartu, Tartu, Estonia; 3 Dept. of Clinical Genetics, United Laboratories, Tartu University Hospital, Tartu, Estonia; 4 Finnish Institute for Health and Welfare, Helsinki, Finland; 5 Department of Clinical Genetics, Section Community Genetics & Amsterdam Public Health Research Institute, Amsterdam University Medical Centre, Location VUmc, Amsterdam, The Netherlands

## Abstract

Genetics has traditionally enabled the reliable diagnosis of patients with rare genetic disorders, thus empowering the key role of today’s clinical geneticists in providing healthcare. With the many novel technologies that have expanded the genetic toolkit, genetics is increasingly evolving beyond rare disease diagnostics. When placed in a transition context—like we do here—clinical genetics is likely to become a fully integral part of future healthcare and clinical genetic expertise will be required increasingly outside traditional clinical genetic settings. We explore transition effects on the thinking (culture), organizing (structure), and performing (practice) in clinical genetics, taking genetic healthcare in Estonia, Finland, and the Netherlands as examples. Despite clearly distinct healthcare histories, all three countries have initially implemented genetic healthcare in a rather similar fashion: as a diagnostic tool for predominantly rare congenital diseases, with clinical geneticists as the main providers. Dynamics at different levels, such as emerging technologies, biobanks and data infrastructure, and legislative frameworks, may require development of a new system attuned with the demands and (historic) context of specific countries. Here, we provide an overview of genetic service provisions in Estonia, Finland, and the Netherlands to consider the impact of historic and recent events on prospective developments in genetic healthcare.

## Introduction

Clinical genetics is an established healthcare service dedicated to the study, treatment, and counseling of individuals with heritable diseases and disorders predisposition. New technologies and methodologies like next-generation sequencing (NGS) technologies, genome-wide association studies (GWAS), and the Clustered Regularly Interspaced Short Palindromic Repeats-associated nuclease 9 (CRISPR/Cas9) system are rapidly expanding the genomic toolkit and increasing the diagnostic power, risk assessment, and treatment options for many diseases, beyond rare disorders, and outside of the traditional boundaries of clinical genetics. While the focus is often on the potential of these technologies for solving persistent challenges for rare diseases—e.g., optimizing diagnosis and characterization—we aim here to explore how such emerging technologies affect the context in which clinical genetics operates.

Healthcare systems are under increasing demands from a changing society and face rising costs, growing fragmentation, and staff reduction (Johansen et al. 2018). Globally, inequalities are widening, the population is aging, and individualism (prioritizing personal interests over those of the wider group) is increasing (Kerry et al. 2017; Santos et al. 2017). At the same time, innovation and new technological developments are continually translated into practice, creating new opportunities for diagnosis, treatment, and care. Medical innovations often inspire their developers to reflect on potential healthcare transformations (Haghi et al. 2017; Hamet and Tremblay 2017; Rus and Tolley 2015; Stark et al. 2019); however, the reality is that the dynamics of healthcare transitions are generally more complex than anticipated.

Emerging technological and societal developments warrant a reconsideration of how clinical genetic services are provided (Battista et al. 2012; Unim et al. 2019). Future genetic healthcare is often envisioned as more proactive—personal, preventive, participatory—and less reactive—diagnose-and-treat (Veltman et al. 2013). Identifying individuals with a high genetic risk for chronic diseases is expected to help target preventive measures, thus postponing or even preventing disease cases and improving quality of life. Crucially, this would also significantly reduce societal and healthcare costs (Inouye et al. 2018; Stark et al. 2019). However, these kinds of proactive genetic-based models have difficulty setting foot in the current healthcare landscape. Various challenges have been identified (e.g., professional protectionism, suboptimal levels of professional genetic knowledge and skills, high standards for evidence, and need for (data-)infrastructure and resources), which could be collectively described as a “lack of preparedness of the healthcare system” (Battista et al. 2012; Martin et al. 2009; Unim et al. 2019).

Putting genetic developments into a transition management context may help in understanding how the changes within healthcare systems could impact the culture, structure, and practice of clinical genetics (Rotmans et al. 2001; Geels and Schot 2010; Loorbach et al. 2017; Rotmans 2005; van Raak 2016; Wittmayer et al. 2017). Typically, transition management is aimed at responsible implementation of change through systematic planning, organizing, and application of key steps in desired transitions. To study key steps of transitions, (aspects of) transition theory can be applied, enabling understanding of the current situation and expected and desired actions. Transition theory has been successfully applied to unravel, structure, and support transitions in other societal systems, such as energy, mobility, and waste transitions (Loorbach et al. 2017). Moreover, a transition-based framework has previously been used to analyze and understand key barriers and facilitators for innovation in specific genetic services (Holtkamp et al. 2017; Rigter et al. 2014). Here, we utilize a transitional framework to consider the impact of historic, recent, and future developments in genetic healthcare.

In the context of this JoCG special issue on rare diseases, we focus on the fields that are currently most relevant for rare disease genetics and where changes in service delivery are observed or expected, i.e., clinical genetics, public health genomics, and genetic research. In the remainder of this contribution, we collectively refer to these as “genetic healthcare.” We first describe the development and key characteristics of genetic healthcare in three countries—Estonia, Finland and, the Netherlands—all of which are dedicated to investing in innovation in healthcare, but differ in the organization and funding of their (genetic) healthcare services (Keskimaki et al. 2019; Kroneman et al. 2016; Lai et al. 2013; Postelnicu 2019). We will subsequently identify the similarities and differences of the clinical genetic regimes in the three countries, characterize key elements of culture, structure, and practice in clinical genetics, and envision how this may influence future developments.

## A multi-level perspective on the origin and development of genetic services in Estonia, Finland, and the Netherlands

**Fig. 1:**
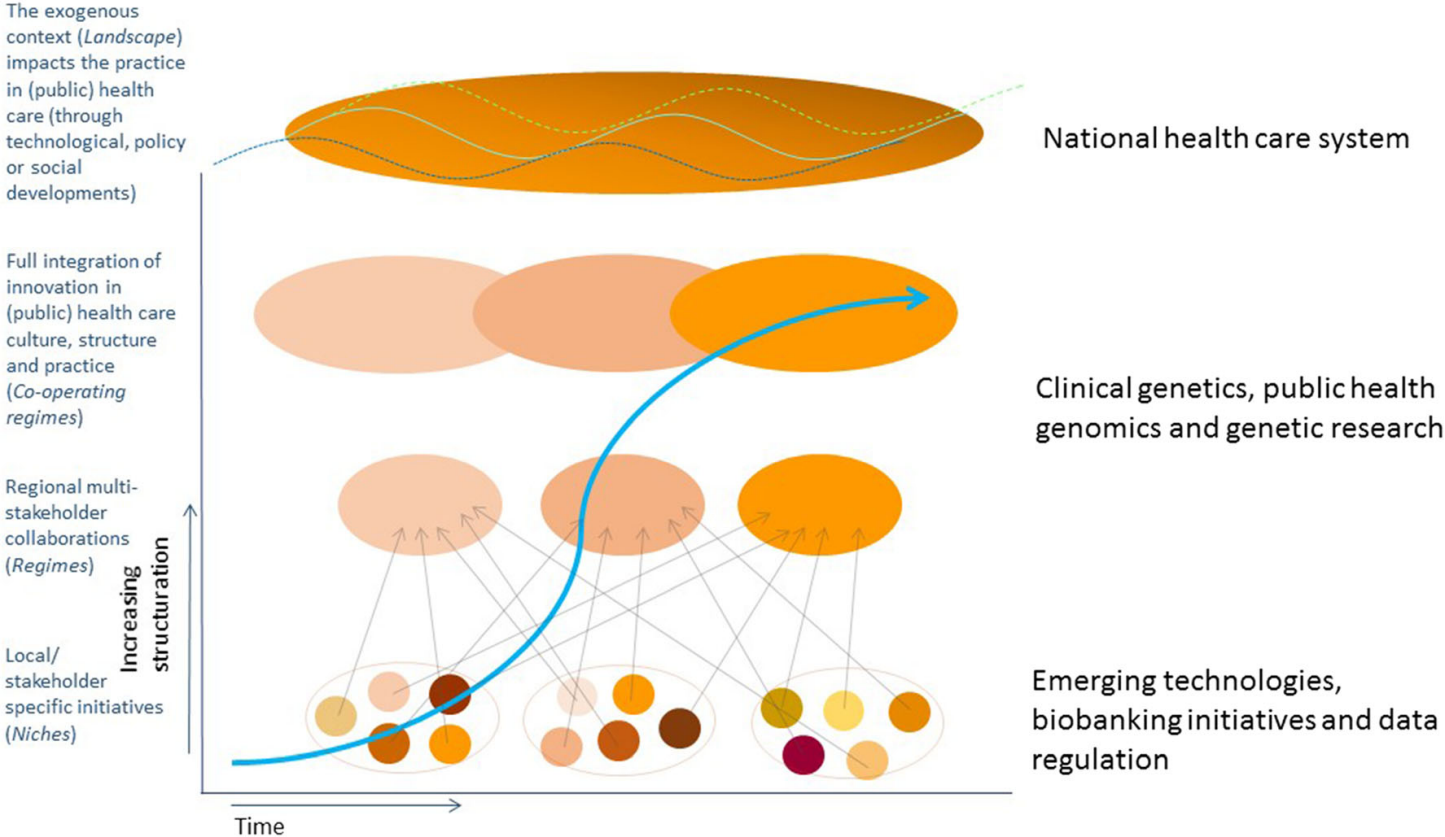
Transition theory. True integration of innovation in (public) healthcare requires different phases in transition and different levels of structuration. While dynamics in the healthcare system (here considered the *landscape*) impact culture, structure, and practice of relative autonomous genetic *regimes*, relevant *niche* developments likely will push towards transition, e.g., by broader implementation, increasing collaboration, and harmonization and structuration of initiatives (adapted from: Rigter et al. 2020)

Following classic transition management theory for socio-technical regimes, we consider genetic healthcare from a multi-level perspective, aiming to unravel and characterize the autonomous developments (“landscape”), the system-specific configuration (“regime”), and the emerging innovations (“niches”) that shape genetic healthcare (Rotmans et al. 2001). Our focus is on the particular configurations in which genetics serves as an application. We will characterize the genetic healthcare regimes as configurations of the elements culture, structure, and practice (Rigter et al. 2014; van Raak 2016). In this context, the national healthcare system may be considered “landscape,” while clinical genetics, public health genomics, and genetic research may serve as distinct “regimes.” Developments in sequencing technologies, biobanking, and data regulation would then be examples of emerging “niches.” An overview of how we apply transition theory is depicted in Fig. 1.

Globally, genetic healthcare emerged in the 1970s following the scientific breakthroughs in the late nineteenth century and the mid-1900s (Hardy 1908; Mendel 1941; Tjio and Levan 1956; Weinberg 1909). How genetic services subsequently developed was predominantly based on the existing healthcare infrastructures shaped by different legislative frameworks. To gain more insight into the impact of nation-specific historical landscapes on current clinical genetic practices, three distinct European countries were selected: Estonia as a Baltic state and a previous member of the Soviet Union, Finland as a representative of the Northern European or Scandinavian countries, and the Netherlands as a representative of western Europe. By the 1970s, healthcare in Finland and the Netherlands had matured into accessible, highly regulated, and government supported systems, while general access and financing of healthcare was limited in Estonia—then still a Soviet republic. To provide insight into the *landscapes*, we first highlight the relevant national policies, regulations, and resources within the three healthcare systems that have predominantly shaped the *regime*s or models for delivery of genetics services in these countries (Battista et al. 2012; Unim et al. 2019). Furthermore, we describe the national clinical genetics, public health genomic, and genetic research *regimes* for each country.

### Estonia

Estonia regained independence from the Soviet Union in 1991 and its current healthcare system *landscape* therefore is considered relatively young (Optimity Advisors 2018). Like the other Baltic states (Latvia and Lithuania), Estonia initiated heavy reforms to break with the centralized, inefficient, and low-quality “Semashko” heritage for healthcare (Sheiman et al. 2018; van Ginneken et al. 2012). Estonia’s government made family medicine a specialty with corresponding postgraduate training (strengthening of primary care), improved the technical proficiency of hospitals (capacity building), and performed a rapid transition from budget-based healthcare to centralized solidarity-based insurance healthcare backed by the Estonian Health Insurance Fund (insurance medicine) (Lai et al. 2013). Healthcare in Estonia is predominantly centrally funded from a proportional flat rate social tax paid by employers. Two reforms—broad scale digitalization in healthcare and the hospital reform—have made the Estonian healthcare systems one of the most progressive in the world (van Ginneken et al. 2012). The focus on technical proficiency in hospitals coincided with the government’s ambition to make Estonia a largely digitalized society. A key outcome of this convergence was the nationwide introduction of Electronic Health Records in 1998, the first country in the world to do so (Tiik and Ross 2010). A number of parallel initiatives—e.g., e-citizenship, e-governance, and e-voting—have further boosted the digital infrastructures and general participation of citizens. Personal use and access of electronic health records by citizens in Estonia is currently the highest rate in Europe (Ćwiklicki et al. 2020). Centrally managed E-Health Database includes Healthcare Imaging Database (established 2006), e-Prescriptions (2010), and e-Registration (2019). Ninety-nine percent of prescriptions are digital and 100% healthcare billing is digital (e-Health Records — e-Estonia (e-estonia.com) n.d.).

The Estonian government is also investing in the realization of personalized medicine at the national level, with an initial focus on pharmacogenomics and application of genetic testing for common complex disorders. For this purpose, there is increased attention to more structured databases, real-time and customized access to health data, and medical decision-support systems (Lai et al. 2013). Related updates to the electronic health records have been announced (Meditsiini Uudised 2020).

The clinical genetics *regime* in Estonia is largely centralized; two clinical genetic departments in Tallinn and Tartu provide access to advanced genetic counseling and cascade screening. Since the end of 1990s, clinical genetics training in Estonia has had its own curriculum, separate from other medical specialties. In general, clinical geneticists work with a broad spectrum of suspected genetic disorders, from rare pediatric problems to more frequent familial cancer syndromes or risk factors for, e.g., thrombophilia. Specialists outside of clinical genetics, for example neurologists, have a long tradition of directly requesting genetic testing for their patients independent of clinical geneticists. In line with centralized clinical genetics services, genetic testing in Estonia is mostly provided by Tartu University Hospital, although genetic testing can also be performed by private laboratories. Clinicians are free to choose service providers. Clinical genetic service is fully reimbursed by Estonian Health Insurance Fund (EHIF), with the volume depending on the allocated budget for specific clinical service providers. Genetic services outside Estonia are also covered but on a case basis via a specific application process, if such services are not available in Estonia.

Maternity and child care services, including aspects of the public health genetic care *regime*—e.g., newborn and prenatal screening—are free of charge, as is the national vaccination program for 12 recommended diseases and cancer screening programs. Neonatal screening covers neonatal hypothyreosis, and 20 treatable metabolic disorders and it is offered to all children since all children automatically have a health insurance (Mikselaar et al. 1998; Reinson 2018). Breast and ovarian cancer screening as well is centrally funded, through the EHIF and coordinated by the Cancer Screening Foundation. Starting from 2021, public cancer screening programs are freely available for all Estonian residents independent of their health insurance status (BNS/TBT Staff (Baltictimes.com) 2020).

The genetic research *regime* in Estonia is profiting from the large genetic dataset that is available in the country. The Estonian Biobank has invested in recruitment and integration strategies which have produced a robust biobank currently representing over 20% of the adult population (Leitsalu et al. 2014). All participants in the Estonian biobank (>200,000) have been genotyped, and a considerable part has been sequenced (5000 exomes and whole genomes). Selected findings (actionable high-risk findings, polygenic risk scores, pharmacogenetics data, etc.) are offered to participants included in various studies. Data use and privacy protection of the biobank, which is linked to data from the Electronic Health Records, is largely regulated by the Estonian Human Genes Research Act (implemented in 2000) and the European General Data Protection Regulation (GDPR) (Keis 2016). The Human Genes Research Act aims to, e.g., ensure the voluntary nature of gene donation and the confidentiality of the identity of gene donors, and to protect persons from misuse of genetic data and from discrimination based on interpretation of the structure of their DNA and the genetic risks arising therefrom. The GDPR is aimed at the protection of natural persons with regard to the processing of personal data and on the free movement of such data in general.

### Finland

The Finnish healthcare *landscape* reflects the Nordic tradition of investment in the quality, equality, and solidarity of healthcare, and provides comprehensive (public) healthcare services for all inhabitants, as regulated by the Constitution (Ahola-Launonen 2016; Keskimaki et al. 2019). Healthcare in Finland is largely financed by taxes (municipalities), insurance fees (National Health Insurance, NHI), and joint employer-employee premiums (occupational healthcare). Publicly funded healthcare is complemented by a growing private healthcare industry due to the increasing demand for various healthcare services (Keskimaki et al. 2019; Finnish Ministry of Social Affairs (Private health care - Sosiaali- ja terveysministeriö (stm.fi) (n.d.)).

The introduction of a clinical genetic *regime* in the 1970s was driven by the convergence of two phenomena (Norio et al. 1973; Reijo Norio 2003b). First, there was increasing evidence that particular rare hereditary diseases were overrepresented in Finland—the so-called Finnish Disease Heritage (FDH) (Reijo Norio 2003a). This led to extensive research on the natural history, treatment, and genetic background of nearly 40 monogenic diseases prevalent in the country (Findis (The Finnish Disease Database: FinDis.org) n.d.). Second, university hospitals founded cytogenetic laboratories, providing genetic diagnostic services for patients with rare diseases (Von Koskull and Salonen 1997). Today, clinics primarily purchase tests from their own university hospitals, with the flexibility to turn to laboratories elsewhere (Finland or Europe—both academic and private) in case of unavailability (Pohjola et al. 2016). Ordering and/or explaining results of genetic tests are not regulated in Finland except that, as for all tests, they have to be ordered by a healthcare professional. The focus of clinical genetics has been on diagnosis and counseling of patients with rare diseases, but the demand for genetic services for more common diseases such as familial cancer is growing. Clinical geneticists focus on diagnostics and cascade screening. Often, the diagnostic tests have already been done by other specialties and the patient is referred for help in interpreting the results, counseling, and family member testing.

As for the genetic research *regime,* human genetic studies in Finland are increasingly facilitated through biobanks. The Finnish Biobanks comprise samples, health information, and genomic data from over 400,000 donors (Finngen (Finngen research project) n.d.). Legislation is largely focused on the collection and use of genetic material within these biobanks, predominantly through the Biobank Act. This legislation is soon expected to be supplemented with the Genome Act, which is intended to enable the use of genomic biobank data in personal healthcare and prevention (Soini 2016)

A hallmark of Finish healthcare—as for other Nordic countries—is high-quality national health registries which provide a solid basis for the national public healthcare *regime*. The registries are established and regulated by specific laws; no consent is required to add personal data to these registries, but access to the data is guarded by detailed processes for all stakeholders.

Many public health services are provided as national programs, largely directed towards health promotion and disease prevention (Healthcare in Finland 2013). These are generally nationally defined, funded, and coordinated while organized at the level of municipalities, comprising various vaccinations and screening programs, including fetal and neonatal screening, as well as maternity and child healthcare. A unique feature emphasized in The Primary Health Care Act (1999) is to mandate the municipalities to take health into account as part of other policies, for instance community planning, and to be responsible for disease prevention and health promotion (Eloranta and Auvinen 2015; Keskimaki et al. 2019). Furthermore, patients and patient organizations play a key role in shaping the genetic services in Finland. Patient organizations actively collaborate with the national healthcare system, offering extra services like organized peer support, rehabilitation services, and specific information on rare diseases (Von Koskull and Salonen 1997). The appreciation for genetic research is also reflected in the public attitude. Patients are aware of their rights (defined in legislation for 30 years) and usually very positive towards population level approaches and medical research (Borodulin et al. 2018).

### The Netherlands

In a transition context, healthcare in the Netherlands is offered in the *landscape* of a predominantly curative system, which has developed from the constitutionalizing of the monarchy in 1848—and consequential shift of power (also of healthcare) to Parliament and (local) governments. It is comprised of various autonomously evolving *regimes* (e.g., the “medical specialists”) (van Raak and de Haan 2018). One of the main characteristics of the Dutch system is the gatekeeping principle, whereby hospital and specialist care require referral from a primary care provider such as a general practitioner, midwife, or dentist, with the exception of emergency care. The 2006 healthcare reforms introduced a market-oriented healthcare system, allowing citizens to freely choose insurer and healthcare provider, while obligating insurers to accept all applicants for the basic benefit package (set by the government), and compete on price and contents of the premium packages (Maarse et al. 2016). Healthcare providers likewise compete for patients and negotiate with insurers on services and fees. The basic health insurance package and compensations for lower incomes protect citizens against catastrophic spending. Out-of-pocket payments are low from an international perspective (Kroneman et al. 2016).

As for the clinical genetic *regime*, clinical genetics has been recognized as a medical specialty since 1987 (Battista et al. 2012). Accordingly, the Ministry of Health has appointed nine dedicated “clinical genetic centers” embedded in academic hospitals, installed legislation prohibiting non-academic hospitals and private companies from providing clinical genetic services (the Specialist Medical Practice Act), and included clinical genetic testing and counseling in basic health insurance (Borry et al. 2012; Nelis 1999). As such, clinical genetics was positioned into a tertiary care service model, evolving into a federated public, mainly diagnostic, support platform for secondary medical specialties such as pediatrics, cardiology, neurology, and oncology (Niermeijer 2011). The strong regulatory and financial embedding of clinical genetics in the Netherlands, however, has created a precedent for Dutch clinical geneticists to be the “treating physician” for their patients (hence often requiring referral from primary or secondary care professionals) at least until the end of the diagnostic odyssey and/or referral to secondary/primary care for treatment. Similarly, the clinical genetics centers have in-house laboratories which perform the majority of genetic testing. Relatively recently, like in Finland, other secondary medical specialties (e.g., oncology) are increasingly ordering genetic tests from the clinical genetic laboratories for their patients, for diagnostics, and personalized treatment plans.

The public healthcare *regime* in the Netherlands is mostly a nationally coordinated healthcare service comprising vaccination, environmental safety, and lifestyle intervention programs (Jambroes et al. 2013). National public health programs in the Netherlands are upon directive from the Ministry of Public Health, Welfare, and Sports (VWS). Scientific insights have traditionally informed public health policy and provision, with patient organizations regularly fueling debate about hypes and hopes of innovations. The national public health services provide various population screening programs (e.g., prenatal, neonatal, breast cancer) and a national immunization program. Besides the national services, public health is delivered as prevention programs at the municipal (for selective and indicated prevention of, e.g., diabetes and obesity) and/or primary care level (for collective prevention of, e.g., cardiovascular risk), but with little attention to genetic factors (Dutch National Institute for Public Health and the Environment (2010); Dutch National Institute for Public Health and the Environment (2021)).

Fueled by the 1989 Health Council report on genetic diagnostics and gene therapy—which included a recommendation to increase the “knowledge and insight in hereditary disease”—the clinical genetics community in the Netherlands started building a solid research infrastructure, resulting in the discovery of (additional) genetic causes for many rare congenital syndromes and the unravelling of novel disease mechanisms. These in turn have fueled the development of therapeutic strategies, and the launch of various large-scale population studies (Aartsma-Rus et al. 2009; Boomsma et al. 2014; Health Council of the Netherlands 1989; Gilissen et al. 2014; Harakalova et al. 2012; Hofman et al. 2007; Hoischen et al. 2010; Rook et al. 1999; Ropers and Hamel 2005; Scholtens et al. 2014; Smeets et al. 1992; Tessadori et al. 2018; Verkerk et al. 1991; Vissers et al. 2004). A number of ongoing, mainly local, cohort studies have recently started collecting and analyzing genetic data from their participants (Boomsma et al. 2014; Hofman et al. 2007; Scholtens et al. 2014).

Most healthcare providers in the Netherlands use a form of electronic patient records. The national roll-out of an electronic patient record system to interconnect these practice-based systems has not yet succeeded, mainly for reasons of privacy. Collecting and studying clinical, including genetic, data is challenging because of the lack of harmonization between the different systems, both from clinical and study cohorts (Kroneman et al. 2016). Recent initiatives aim to improve the findability, accessibility, interoperability, and reusability (the FAIR-data principle) of genetic data in the Netherlands (Zorginstituut Nederland, 2018).

## Characterizing the *regimes* of genetic healthcare—culture, structure, and practice

Developments in *landscapes* have largely determined the characteristics of the current *regimes* of clinical genetics, public health genomics, and genomic research in Estonia, Finland, and the Netherlands. Here, we provide a more in-depth analysis of the similarities and differences of the clinical genetic regimes in these three countries in terms of the key elements of culture (how we think), structure (how we organize), and practice (what we do) (Table 1).

**Table 1:** Overview of clinical genetics in Estonia, Finland, and the Netherlands in terms of the key elements of culture (how we think), structure (how we organize), and practice (what we do).

Level	Component	Estonia	Finland	The Netherlands
Healthcare system (*landscape*)	Accessibility and financing	Public (private as a choice), mainly financed through fund by employers Largely insurance-based payment	Public (private as a choice), through fragmented health financing arrangements (by municipalities, insurance, employers, and households) Largely publicly funded, with relatively small patient fixed fees	Mainly public through insurers, with market-based pricing Largely insurance-based payment (as basic insurance is obligatory)
Genetic services (*regimes*) - culture	Goal of clinical genetics	Diagnostics based on clinical symptoms, with increasingly wider and pre-symptomatic screening with whole exome/genome sequencing for the benefit of the individual as well as for research purposes.	Diagnostics based on clinical symptoms; early use of panels and whole exome sequencing increasing. Plans to extend use of genomic data for other healthcare goals of the individual as well as for research (according to the present draft Genome Act)	Diagnostics based on clinical symptoms, mainly targeted genetic testing to confirm diagnosis. Early use of panels and whole exome sequencing increasing for some indications (when proven cost-effective or for research purposes). Plans to study impact of use of genetic data for other healthcare goals.
	Roles and responsibilities in clinical genetic testing and counseling	Clinical geneticists primarily focus on diagnostics and cascade screening. To a limited extent, genetic testing is ordered by other specialty doctors beyond clinical geneticists. Counseling performed by clinical geneticists	Clinical geneticists primarily focus on diagnostics and cascade screening. Diagnostic testing as well as informing about the results is also done beyond genetics clinics. Counseling performed by clinical geneticists and genetic nurses (without specific training programs for genetic counseling)	Clinical geneticist as “treating physician.” In addition, other specialists who have subspecialized in genetics (e.g., onco-geneticists) increasingly order and counsel genetic testing at clinical genetic laboratories. Counseling performed by clinical geneticists and specifically trained genetic counselors
	Patient attitude	Patient attitude is positive for genetic testing in general and towards biobanking.	Patients are aware of their rights (already defined in legislation for 30 years) and usually positive towards population level approaches and medical research.	Individual autonomy and informed decision-making are key values in all genetic services.
Genetic services (*regimes*) - practice	Genetic testing & screening	Genetic tests purchased from best available source (including commercial laboratories); panels and whole exome sequencing are increasingly favored. Practices undergoing transition and clinical pilots for pre-symptomatic screening for monogenic disorders are introduced through biobank participants (Alver et al. 2019; Leitsalu et al. 2020) Population screening for some hereditary disorders through publicly funded and coordinated (cancer, newborn, and prenatal) screening programs	Genetic tests purchased from best available source (including commercial laboratories); panels and whole exome sequencing are increasingly favored. Practices undergoing change, mainstreaming of genetics is happening and clinical pilots for pre-symptomatic screening for complex disorders are planned in biobank participants Population screening for some hereditary disorders through publicly funded and coordinated (cancer, newborn, and prenatal) screening programs	Genetic testing generally confined to clinical genetic lefts; mainly targeted sequencing approach with whole exome sequencing/whole genome sequencing increasingly applied for selected patient groups Practices adhering to dynamics, mainstreaming is slowly happening and studies on impact of clinical genetic pre-symptomatic screening are initiated locally Population screening for some hereditary disorders through publicly funded and coordinated population (cancer, newborn, and prenatal) screening programs
Genetic services (*regimes*) – structure	Organization	2 clinical genetic departments (Tartu, Tallinn) National electronic health records, health registries, centralized laboratory services, foreign testing available, and reimbursed upon need. Public health centrally coordinated with local service provision. Biobanking involving 20% of adult population with data from genome-wide arrays and whole genome sequencing.	5 clinical genetics lefts + a small one for Swedish speaking minority; some private clinical geneticists. Laboratory services purchased from various sources including abroad, often university hospital laboratories. Public health centrally coordinated with local service provision. Biobanking aiming at 10% of the population by 2023 with standard genome wide arrays of all and whole-exome/-genome sequencing of part of the samples; at present only for research purposes National Health Registries	9 clinical genetic lefts, each clinical genetic left autonomously builds sequencing and data infrastructure. Public health centrally coordinated with local service provision. Local—sometimes linked—biobanks, but no national infrastructure.
	Legislation/regulation	Estonian Human Genes Research Act (2000) General Data Protection Regulation (2018)	Act on the Status and Rights of Patients (1992) Biobank Act (2012) General Data Protection Regulation (2018)	Healthcare Professionals *Act* (1987) Exceptional medical procedures Act (1978) General Data Protection Regulation (2018)
	Electronic health records and data-exchange	Linking biobank with electronic health record, but limited data transfer from biobanking to healthcare yet	Local electronic health record systems interacting via National Patient Data Repository which enables citizens to partly see and control their data. At present, no connection to biobank data	Local electronic health record systems, increasingly exchanged between healthcare providers

### Culture

Traditionally, the purpose of genetic healthcare (particularly clinical genetics) has been to provide diagnoses for predominantly rare diseases. However, in both Estonia and Finland, genetic diagnostics have been considered part of the tool set of medical specialists other than clinical geneticists (e.g., pediatricians). This aspect of culture, namely the vision on the main application of genetics for diagnostics and perceived need to confine genetic services to the clinical genetic specialty, may affect attitudes towards adoption of emerging new technologies (*niches*) beyond the traditional boundaries of clinical genetics. For instance, the first attempts to use results from research on biobank samples in preventive healthcare have already been undertaken in Estonia, e.g.. return of copy number variants and actionable monogenic findings related to familial hypercholesterolemia and hereditary breast and ovarian cancer (Alver et al. 2019; Leitsalu et al. 2016, 2020). By the end of 2019, over 2500 participants had received a personal report with polygenic risk scores on type 2 diabetes, cardiovascular disease, early onset menopause, thrombophilia, hypolactasia, and glaucoma, as well as pharmacogenetics data. In Finland, similar application of polygenic risk scores to health promotion is being piloted in research settings (Marjonen et al. 2020). Some local genomic initiatives have only just initiated in The Netherlands, to apply broader genomic pre-symptomatic testing beyond the setting of clinical genetics (Sedaghati-Khayat et al. 2020)^,^(Amsterdam UMC, Locatie VUmc - Mijn DNAmedicatiepas n.d.).

There is an ongoing debate on the information generated and reported from diagnostic studies in clinical genetic services. Genetic laboratories in Europe have traditionally followed the recommendations of the European Society of Human Genetics to apply targeted approaches in order to minimize incidental or secondary findings (Isidor et al. 2019; Matthijs et al. 2016; Vears et al. 2018). There is an increasing call for applying broader approaches for reasons of cost-effectiveness, opportunities to re-analyze or re-interpret (Sun et al. 2015), or as contributions to research (van El et al. 2013). Also, the opportunity to utilize data generated from high throughput sequencing to inform patients about “actionable variants” beyond the primary clinical inquiry (often referred to as “opportunistic screening”) is highly debated. The European Society of Human Genetics remains reluctant in this context and recommends a “cautious approach” (de Wert et al. 2020). Clinical genetic centers in Europe therefore still tend to prefer targeted interpretation approaches (Pajusalu et al. 2018; Vrijenhoek et al. 2015; Weiss et al. 2013). Conversely, the philosophy behind the Estonian Biobank has always been to collect the broadest possible genotypic data into a population biobank and use these data for healthcare whenever appropriate (Leitsalu et al. 2015). In Finland, biobanks linked with national healthcare registries have been collected to form a powerful national infrastructure for research and the use of Biobank data also in healthcare and health promotion is being formalized.

### Structure

Clearly, clinical genetics has been the main *regime* for genetic healthcare applications in all three countries. However, organization differs considerably. For instance, in the Netherlands, the nine clinical genetic centers are autonomous units, devoid of competition, with essentially all necessary resources (finance, infrastructure, staff) available in-house. Dutch law prevents non-academic hospitals and private industry from conducting genetic services, while in Estonia and Finland, these roles are less confined. In Estonia and Finland, clinical genetic services offer genetic counseling and use both the hospitals’ as well as external (including foreign) laboratories for genetic testing, services increasingly also requested by non-genetic medical specialists. Besides the generally recognized medical profession of clinical geneticists in most countries in Europe, the three countries described here differ in their training and deployment of professionals specifically trained to conduct genetic counseling: genetic counselors. While Estonia and Finland lack specific training programs for this profession, Finland does have nurses trained in genetics. The Netherlands has several training programs and over 50 genetic counselors (Abacan et al. 2019).

In all three countries, there are indications of an increasing burden on clinical genetics staff and budget, predominantly as a result of increasing demand (Lynch and Borg 2016). At present, there are dedicated, often registered, clinical genetics staff (i.e., clinical geneticists, laboratory specialists, or genetic nurses and/or counselors) in all three countries (Lynch and Borg 2016). However, based on growing demand and waiting times for the clinics, there appears to be a general need for more genetically literate medical staff. Central organization—such as in Estonia—may require a relatively small staff. In Finland, long geographical distances require clinics in many parts of the country, and thus a considerable staff even in the sparsely populated northern and eastern parts. The federated clinical genetic *regime* (centrally coordinated, but with distributed responsibilities) in the Netherlands, with clinical genetics as an established department in each of the eight academic hospitals—thus boosting the demand for clinical genetic services from other medical specialists—would probably need the most increase in staff. A revolutionary change in the working traditions from comprehensive face-to-face counseling to, e.g., (online) tutorials supplemented, when needed, with short counseling sessions would radically decrease the need for more personnel. On the other hand, clinical geneticists or genetic counselors/nurses are increasingly needed as consultants and advisers outside the clinical genetic centers, as the use of genetic information expands in clinical practice and only patients with complex findings are referred to clinical geneticists. Still, clinical geneticists are the only specialty systematically engaging family members for cascade screening.

Whereas most of the legislation for clinical genetics is country-specific, the recent introduction of European legislation for the protection of natural persons with regard to the processing of personal data and on the free movement of such data (GDPR) has initiated reflections on the status, management, and use of genetic data across Europe. The effect of this European data uniformization effort has been different across countries. In Finland and Estonia, the traditional “broad consent model” in use when collecting biobank samples, or “no consent” relating to Finnish national healthcare registries, are being complemented by strict regulation of data access and the rights of donors/patients in specific national legislation, to be in line with GDPR (Soini 2016). Conversely, in the Netherlands, legislation around personal data traditionally seeks extensive control and assurance, e.g., through detailed consent procedures. The explicit procedures in the Netherlands on secondary use of data, unsolicited findings, and re-contacting therefore generally facilitate GDPR implementation, despite the variety of consent procedures across the country (Vrijenhoek et al. 2015). All other aspects of genetic services in the Netherlands are regulated by a patchwork of laws, codes of practices, and other ethical instruments, such as the Act on Population Screening (Wbo). These are converted into guidelines by national professional organizations, e.g., for clinical geneticists and for genetic laboratories (Vereniging Klinisch Genetische Laboratoriumdiagnostiek n.d.; Vereniging Klinische Genetica Nederland n.d.).

The absence of equivalents to the Dutch Specialist Medical Practice Act in Finland and Estonia (and in many other countries in Europe) more easily allows not only other specialists but also various local health centers, regional hospitals, and private clinics to offer genetic services. This potentially facilitates more efficient implementation of new genetic innovations outside the traditional clinical genetic *regime*, such as pre-symptomatic (pharmacogenomics) testing and liquid biopsy screening in cancer patients for early diagnosis or personalized treatment.

While biobanks and healthcare data in Estonia and Finland are already largely centralized in national registries, the Netherlands is only recently trying to make fragmented genetic data findable, accessible, interoperable, and reusable (FAIR) (Radboud 2018; Wilkinson et al. 2016).

### Practice

In terms of the services delivered in the clinical genetic *regimes*, there seems to be general agreement on the essential components of clinical genetic care for patients in all three countries. For rare diseases, typically a primary or secondary care specialist refers the patient to genetic specialists, detailed phenotyping and clinical evaluation, pre- and post-test counseling, and possibly cascade screening (Vears et al. 2018). Genetic services for more common variants or diseases (e.g., polygenic risk scores) are generally considered to be (or become) part of routine primary or secondary care. Effective implementation in this setting, however, would require a redesign of these current *regimes* to meet essential prerequisites for good genetic care. This would include more emphasis on medical decision-making, formulating best practices on communicating genetic results and their consequences, and arranging of reimbursement. This is especially true for the countries where, as in the Netherlands, clinical genetic services have traditionally been mainly confined to the clinical genetic centers and clinical genetic specialists.

Since the introduction of high throughput sequencing techniques, the possibility of wider analysis of genomic data in research and clinical care became feasible, and ongoing decreasing cost trends are driving ever-wider implementation. At the present, in the clinical genetic regimes, a shift from a specific test to confirm a clinical diagnosis is seen towards broader tests such as gene panels or whole exome sequencing. This has given rise to several ethical and practical dilemmas; for some time already, the clinical genetics community is struggling to reach a consensus on proper use, including the appropriate model that comprehensively covers consent, informs family members, and provides directions for re-contacting and use of genetic data for research (van El et al. 2013; Vears et al. 2018). Although the European Society of Human Genetics provides recommendations on some of these aspects, practices are generally not harmonized at the national level, let alone the European level.

New service delivery models within the clinical genetics’ *regime* were quickly and broadly implemented in all three countries during the first SARS-Cov2 lockdowns in 2020. E-counseling replaced in-person counseling where needed, and online tutorials and educational materials were used to increase efficiency of information provision. Many guidelines and recommendations for new service delivery models have been published (e.g., on individual face-to-face counseling versus e-information, testing methods, returning results from biobanks, incidental, and secondary findings (Battista et al. 2012; Isidor et al. 2019; Sun et al. 2015; Vears et al. 2018)), but it is unclear what the actual uptake and (especially mid and long term) effects will be.

Population (cancer, neonatal, and prenatal) screening programs are implemented in the public health regimes of all three countries, while to a differing extent, pre-symptomatic screening for other hereditary disorders is implemented, piloted, or studied through the biobanks or other research infrastructures. While Estonia and Finland are inclined to do this at national level, the first broader applications of, e.g., pharmacogenetics or polygenic risk scores in the Netherlands is currently still restricted regionally to initiatives at medical centers.

## Discussing the implications: dynamics influencing transitions in genetic healthcare

Development of truly transformative genetic services requires extensive exploration, preparation, implementation, and evaluation (e.g., considering education, infrastructure, finances), which are generally long-term processes. The timely and careful planning of these processes are therefore of utmost importance to drive transitions that improve genetic healthcare (Rigter et al. 2014). The regime descriptions of Estonia, Finland, and the Netherlands are based on discriminators in culture, structure, and practice that provides routes to the expected or required response to dynamics in relevant “niches.” These dynamics could be technology-driven, considering the quickly expanding genetic toolkit, but also public perceptions, values, and needs in (local) *regimes* or *landscapes* may change after a disruptive event. Perhaps, the public health crisis caused by the SARS-Cov2 pandemic and its potential implications on, e.g., public trust and views on solidarity and autonomy in addition to lessons from application of genomic sequencing technology in this context, could instigate disruption to cause transformation in healthcare. If true, this will likely also affect clinical genetic service provision.

Technological advancements have increased the demand for high throughput sequencing techniques and genetic testing in general for more and more patients. This has raised discussions about the sustainability of the existing clinical genetic framework in many countries. Prioritizing applications and subsequently allocating budgets to the most (cost)effective tests will require evaluation of many different techniques, interventions, and outcomes. For many reasons outside the scope of this paper, this evaluation will be challenging (Love-Koh et al. 2018; Vrijenhoek et al. 2018).

Furthermore, genetic insights have increased the demand to widen the application of genetic testing beyond diagnosing rare genetic diseases. This may possibly introduce broader implementation of novel service delivery models, e.g., “mainstreaming” to non-genetic medical specialists, and pharmacogenetic support for treatment and genetic testing in the context of disease prevention (Rigter et al. 2014). The extent to which this happens—and how—strongly depends on the local culture, structure, and practice: all influencing the acceptability of certain applications. Of the three countries we have presented here, Estonia, with its modern and tradition-free healthcare, is likely best positioned to widely implement medical genomics in healthcare, at least in the short term. Indeed, the first trials to screen biobank participants and disclose results from testing for familial hypercholesterolemia and breast and ovarian cancer risk are already ongoing (Alver et al. 2019; Leitsalu et al. 2020).

Moreover, the role and position of biobanks in genetic research (and resulting care) have and will significantly influence the genetics regime, including applications for rare diseases. Depending mainly on organizational characteristics and general acceptance of use of genetic data, most likely, there will be an increasing application of genetic analysis for healthy individuals which could be appropriate and feasible for prevention-based healthcare. Uptake of these applications is dependent on the existence of national biobank initiatives and legislative prerequisites, public perceptions of genetic data and its associated aspects of access, identity and uncertainty, and the future position of genetics in the healthcare system, and in society as a whole.

In terms of organization of clinical genetic services, genetic counselors/nurses are still deemed best equipped to address the increasing need for counseling, such as risk communication to non-symptomatic high-risk individuals or by facilitating cascade screening. However, the state of genetic counseling as a profession, or the necessary training in different countries across Europe, varies to a great degree, potentially already leading to suboptimal care in some regions (Abacan et al. 2019). In order to provide good genetic healthcare, the clinical genetic workforce needs to be expanded and optimized in order to meet the current demands, both in terms of quality and quantity. Moreover, there is a considerable lack of applicable genetic knowledge beyond clinical genetics, especially when genetic testing is increasingly being applied outside the traditional clinical genetic *regimes*, as expected. In addition, new genetic tools and developments require retraining of existing genetic counselors, and possibly the creation of new medical professions specialized in various advanced medical genetic techniques.

Whereas population screening programs currently are generally the responsibility of public health institutes or other national agencies, the expertise and experience within clinical genetics can be instrumental in the development of current and future pre-symptomatic screening strategies. This was particularly apparent in the implementation of non-invasive prenatal testing (NIPT), where—at least in the Netherlands—clinical geneticists took a central role in the development of counseling, training, and testing strategies (van der Meij et al. 2019; van Schendel et al. 2017). A similar attitude could be envisioned for genetic testing to enable informed decision-making in family planning. Especially, rare disease patient organizations seem to be increasingly requesting comprehensive non-invasive prenatal diagnostics, preimplantation genetic diagnostics, and/or preconception carrier screening programs (Bell et al. 2011; Geraedts et al. 2001; Wright and Burton 2008).

## Conclusion

Dynamics in *niches* are influencing the local culture, structure, and practice of genetic services. More widespread application of genetics beyond traditional clinical genetics has the potential to transform genetic healthcare. However, major obstacles including lack of clinical evidence for testing for polygenic traits, ethical, legal, and social challenges and the need for development of innovative reimbursement models remain.

By effectively adapting to dynamics in both the *landscape* and emerging *niches* locally, but simultaneously attuning developments and sharing experience across countries in Europe, we believe that optimization of genetic healthcare can ultimately be achieved.

The clinical genetic regime should sustainably use their expertise and experience in the context of diagnostics, counseling, and cascade screening for patients and families with rare diseases. Simultaneously, this knowledge could be translated into novel strategies towards service provision and educating other healthcare professionals. Moreover, continuous education and training of current genetic professionals is needed in order to keep up with (technological) developments in the field. Of crucial importance will be data sharing and breaking down the silo mentality, not only within countries but also between nations.
